# Abnormal Characterization and Distribution of Circulating Regulatory T Cells in Patients with Chronic Spinal Cord Injury According to the Period of Evolution

**DOI:** 10.3390/biology12040617

**Published:** 2023-04-19

**Authors:** Ana M. Gómez-Lahoz, Sergio Haro Girón, Jorge Monserrat Sanz, Oscar Fraile-Martínez, Cielo Garcia-Montero, Diego J. Jiménez, Diego de Leon-Oliva, Miguel A. Ortega, Mar Atienza-Perez, David Diaz, Elisa Lopez-Dolado, Melchor Álvarez-Mon

**Affiliations:** 1 Department of Medicine and Medical Specialities, Faculty of Medicine and Health Sciences, University of Alcalá, 28801 Alcalá de Henares, Spain; alahoz1199@gmail.com (A.M.G.-L.); miguel.angel.ortega92@gmail.com (M.A.O.);; 2 Ramón y Cajal Institute of Sanitary Research (IRYCIS), 28034 Madrid, Spain; 3 Service of Rehabilitation, National Hospital for Paraplegic Patients, Carr. de la Peraleda, S/N, 45004 Toledo, Spain; 4 Service of Internal Medicine and Immune System Diseases-Rheumatology, University Hospital Príncipe de Asturias, (CIBEREHD), 28806 Alcalá de Henares, Spain

**Keywords:** chronic spinal cord injury (SCI), regulatory T cells (Treg), period of evolution, immune dysfunction

## Abstract

**Simple Summary:**

Immune dysfunction is a major feature of chronic spinal cord injuries (SCIs). It is associated with many of the complications observed in these patients, such as increased vulnerability to infections and other medical challenges. The role of regulatory T cells (Tregs) after SCI is beginning to be more acutely understood; however, little is known about their implications at chronic stages. By using flow cytometry, we characterized circulating Tregs from chronic SCI patients according to the time of initial injury (1–5 years; 5–15 years; and >15 years). Our results demonstrate significant changes in the immunological phenotypes of these cells, especially in patients with long periods of evolution (5–15 years and >15 years with chronic SCI). Despite the fact that a deeper understanding of the immune dysfunction caused by chronic SCI is still required, the characterization of circulating leukocytes such as Tregs and other populations can open up the possibility of finding translational biomarkers or therapeutic approaches that may aid in the clinical management of chronic SCI.

**Abstract:**

Spinal cord injury (SCI) is a progressive and complex neurological disorder accompanied by multiple systemic challenges. Peripheral immune dysfunction is a major event occurring after SCI, especially in its chronic phase. Previous works have demonstrated significant changes in different circulating immune compartments, including in T cells. However, the precise characterization of these cells remains to be fully unraveled, particularly when considering important variants such as the time since the initial injury. In the present work, we aimed to study the level of circulating regulatory T cells (Tregs) in SCI patients depending on the duration of evolution. For this purpose, we studied and characterized peripheral Tregs from 105 patients with chronic SCI using flow cytometry, with patients classified into three major groups depending on the time since initial injury: short period chronic (SCI-SP, <5 years since initial injury); early chronic (SCI-ECP, from 5–15 years post-injury) and late chronic SCI (SCI-LCP, more than 15 years post-injury. Our results show that both the SCI-ECP and SCI-LCP groups appeared to present increased proportions of CD4+ CD25+/low Foxp3+ Tregs in comparison to healthy subjects, whereas a decreased number of these cells expressing CCR5 was observed in SCI-SP, SCI-ECP, and SCI-LCP patients. Furthermore, an increased number of CD4+ CD25+/high/low Foxp3 with negative expression of CD45RA and CCR7 was observed in SCI-LCP patients when compared to the SCI-ECP group. Taken together, these results deepen our understanding of the immune dysfunction reported in chronic SCI patients and how the time since initial injury may drive this dysregulation.

## 1. Introduction 

Spinal cord injury (SCI) is a severe and progressive neurological condition, often resulting in permanent disability and serious comorbidities [1,2]. Globally, the estimated incidence of SCI ranges from 3.6 to 195.4 patients per million inhabitants, and in the United States alone, the incidence of acute and chronic SCI is more than 10,000 cases per year, with an estimated cost of USD 4 billion annually [3,4]. After the initial trauma (designated as the primary injury), the spinal cord tissue suffers a progressive degeneration (secondary injury), which is often classified into acute (in the first 48 h), sub-acute (until 2 weeks), and chronic phases (which extend from days to years) [5]. The chronic phase is the longest for SCI subjects. Apart from the primary and secondary injuries occurring in the spinal cord, there are plenty of systemic alterations observed in SCI patients, virtually affecting the organs and systems of the entire body [6]. 

Peripheral immune dysfunction is a major concern in patients with chronic SCI [7]. The immune system is recruited to the injury site in the early stages, orchestrating spinal cord repair after the traumatic event and promoting secondary mechanisms of damage [8]. This leads to a persistent, non-self-limiting, inflammatory cascade that is permanently sustained, even in long chronic periods [9]. Concomitantly, there is a loss of neuroendocrine modulation of the immune system and lymphoid structures such as the bone marrow, which explains the immunological changes occurring in these patients [10]. Immune dysfunction can manifest in systemic inflammation, autoimmunity, or immunosuppression, supporting the heterogeneity of immune responses that occur after SCI [8,9,11,12]. Consequently, peripheral immune dysfunction partially explains the high risk of SCI patients suffering from different infections, especially urinary tract infections (UTIs) and pneumonia, which represent two central causes of rehospitalization in this population [13,14,15].

Globally, the immune dysfunction associated with SCI can be studied by observing changes in the different compartments of circulating immune cells of SCI patients, as has been reported in multiple studies [16,17,18]. T lymphocytes are major modulators of adaptative immune responses [19]. Previous works have evidenced that these cells play a pivotal role in the local and systemic changes occurring after SCI [7,20]. Immunologically, T cells can be classified into CD4 and CD8 T cells. Regulatory T cells (Tregs) are a special subset of CD4 T cells that are critically implicated in the suppression of immunoinflammatory responses [21]. Immunophenotypically, Treg cells are characterized by the expression of CD3, CD4, CD25, and FOXP3, but lack CD127 [22]. Other markers, such as CD45RA, CCR7, CCR5, and CCR2, can be used to distinguish between specific Treg subpopulations [23,24,25,26]. Following SCI, the production of damage-associated molecular patterns (DAMPs) and pro-inflammatory cytokines drives the infiltration of T cells in acute stages, and this infiltration is sustained over time [27]. This immune extravasation may exacerbate tissue damage and add to the cascade of subsequent injuries. By inhibiting the activation and operation of other immune cells such as effector T cells and macrophages, Tregs can aid in limiting this response. Indeed, the major role of Tregs in limiting inflammation-dependent damage in the spinal cord tissue has been previously reported [28]. These immunomodulatory roles are achieved due to the production of anti-inflammatory cytokines such as interleukin 10 (IL-10); the cell death promotion of cytotoxic T cells; or the sequestering of different proinflammatory cytokines from the environment, such as IL-2 [29]. In animal models of SCI, studies have demonstrated that increasing the amount of Tregs can enhance tissue healing and functional recovery. For instance, one study discovered that injecting Tregs into the wounded spinal cords of mice reduced inflammation and increased the number of neurons that survived [30]. In addition, Treg cells can favorably influence the function of different glial cells after SCI. For instance, they can promote differentiation and migration of oligodendrocyte precursor cells (OPCs), leading to remyelination by oligodendrocytes; blockade of neurotoxic activation of the astroglia; and enhancing phagocytosis of myelin debris or reducing pyroptosis in microglial cells [29].

Despite the relevance and functions of Tregs that have been established in the spinal cord during the early stages of SCI, little is known about the relevance and functional changes of these cells in the chronic phase. Previous works have demonstrated that patients with chronic SCI present significant alterations in circulating T lymphocytes, including Treg cells [31,32]. However, the extent to which immune dysfunction and circulating Treg populations have been studied in patients with chronic SCI is far from fully understood. Hence, the main purpose of the present work is to deepen the current knowledge of circulating Treg subsets depending on the time since initial injury in patients with chronic SCI.

## 2. Patients and Methods

### 2.1. Study Design 

A prospective study was conducted on 105 patients with chronic SCI and 40 healthy controls (HC). To study the immune system throughout the course of SCI, patients were divided into three subpopulations: the short period (SCI-SP), for those patients with a time of evolution less than 5 years; early chronic (SCI-ECP), for those between 5 and 15 years post-injury; and late chronic (SCI-LCP), if the duration of evolution was above 15 years. 

All individuals were properly informed before enrollment, providing their signed written consent, which was approved by the Institutional Review of National Hospital for Paraplegic Patients (10 September 2015). The present work was developed according to the basic ethical principles of autonomy, beneficence, non-maleficence, and distributive justice, following the Good Clinical Practice guidelines, the principles contained in the most recent Declaration of Helsinki (2013), and the Oviedo Convention (1997). The data and information collected followed current legislation on data protection (Organic Law 3/2018 of 5 December, Protection of Personal Data and Guarantee of Digital Rights and Regulation (EU) 2016/679).

Medical information from the SCI patients was collected in a routine clinical examination in the Physical Medicine and Rehabilitation Department, including the following data: (1) baseline demographic features; (2) time and mechanism of injury; (3) neurologic injury level and related severity; (4) tonic and phasic spasticity; (5) presence or absence of pain, as well as its type and severity; (6) history of prior infections or other indicators of a chronic SCI complication; (7) comorbidities; (8) use of contemporary medications; (9) fatigue; (10) anxiety and depression levels; (11) degree of independence in daily living activities; and (12) self-reported quality of life and health status. 

### 2.2. Sample Collection and Inclusion/Exclusion Criteria

Blood samples were extracted from chronic SCI patients at the time of the medical evaluation in the outpatient clinic area. Blood was extracted through standard venipuncture, using an established aseptic technique for all cases. The inclusion criteria for this study were: (1) age ≥ 18 years; (2) history of any level of SCI ≥ 1 year prior; and (3) SCI with varying severity ranging from grades A to E according to the American Spinal Injury Association (ASIA) Impairment Scale (AIS) [33]. A Physical and Rehabilitation Medicine clinician, board-certified in SCI medicine, evaluated the subjects’ injuries according to the International Standards for Neurologic Classification of Spinal Cord Injury [34,35]. 

On the other hand, exclusion criteria were: (1) a concomitant infection with notable severity, such as a UTI or a respiratory infection, evidenced by a positive culture in the last 3 months; (2) chronic viral or bacterial infection; (3) serious cardiovascular disease (CVD); (4) an autoimmune disease or immunodeficiency; (5) hematopoietic, renal, lung, or hepatic complications; (6) a clinical diagnosis of endocrine or metabolic disorders; (7) a history of cancer; (8) pressure ulcerations in the last year; (9) use of immunomodulatory drugs such as steroids in the last 3 months; (10) malnutrition; (11) being pregnant or in lactation period; and (12) any psychiatric disorder. 

### 2.3. Isolation of Peripheral Blood Mononuclear Cells and Immunophenotype Studies

Peripheral blood mononuclear cells (PBMCs) were isolated using Ficoll Hypaque (LymphoprepTM, Axis-Shield, Oslo, Norway) gradient centrifugation. Then, cells were resuspended in RPMI 1640 (BioWhittaker Products, Verviers, Belgium) supplemented with 10% heat-inactivated fetal calf serum, 25 mM HEPES (BioWhittaker Products), and 1% penicillin–streptomycin (BioWhittaker Products). Cell enumeration was performed by conventional light microscopy in a Neubauer chamber following the trypan blue dead cell exclusion criteria. The viability of PBMCs was assessed by both trypan blue (light microscopy, Primostar 3, Carl Zeiss Iberia, S.L., Spain) and 7-amino actinomycin D (7-AAD) (flow cytometry, Sigma Aldrich, Merck Life Science, Madrid, Spain) exclusion. 

T cells were phenotypically analyzed in PBMC by nine-color polychromatic flow cytometry on a FACSAria cytometer using FACSDiva software (Becton-Dickinson, NJ, USA). For surface staining, 1 million PBMC cells in 4 FACS tubes were incubated with combinations of fluorescein isothiocyanate (FITC)-anti-CCR2/CCR5/CCR6 monoclonal antibodies (Moabs) (Biolegend, San Diego, CA, USA), peridinin chlorophyll protein (PercP)-anti-CD3 (Biolegend), phycoerythrin-cyanin seven (PE-Cy7)-anti-CD25 (BD), allophycocyanin-alexa-780 (APC-Alexa780)-anti-CCR7 (eBioscience, San Diego, CA, USA), brilliant violet-405-anti-CD4 (Biolegend), and brilliant violet-605-anti-CD45RA (Biolegend).

For all samples, once the MoAbs were added, the cells were incubated for 20 min at 4 °C in the dark. Afterward, cells were washed in phosphate-buffered saline (PBS) to remove excess antibodies, and 100 µL of PBS was added for subsequent acquisition by flow cytometry. Control studies with unstained cells and cells incubated with isotype-matched irrelevant MoAbs were performed for each experiment. In a forward-scatter–side-scatter dot plot (FSC-SSC), a biparameter gate was drawn around the lymphocyte population. Analyses were carried out using FlowJo 10.00 software (TreeStar Inc., Ashland, OR, USA).

### 2.4. Statistical Analysis 

Nonparametric Mann–Whitney U tests were applied to compare the chronic SCI patients and the HCs. All calculations were carried out using the Statistical Package for the Social Sciences (SPSS, version 22.0, Chicago, IL, USA). Significance was established at *p*-values (*p*) < 0.05 (*), *p* < 0.01 (**), and *p* > 0.001 (***). Data are presented as medians (interquartile range).

## 3. Results

### 3.1. Patients Demographics

We studied 101 patients (mean age: 35.23 ± 12.84 years; 70.10% men) with chronic SCI and 40 HCs (32.74 ± 8.92 years; 63.70% men). The mean time of SCI onset was 12.99 ± 9.16 years.

The neurological spinal damage was located within C1–C4, C5–C8, T1–T6, T7–T12, and the lumbosacral metamers in 23.8%, 20%, 26.27%, 20.95%, and 8.57% of the patients, respectively. In other words, more than 70.40% of our patients had an SCI above T6. Concerning the ASIA, 46.67% of the patients were AIS A, 16.19% of the patients were AIS B, 16.19% of the patients were AIS C, and 20.95% of the patients were AIS D, indicating that despite 79.04% of the patients exhibiting incomplete lesions, only 62.85% reported incomplete motor injuries, with different extents of intralesional motor preservation and theoretically better mobility profiles.

### 3.2. Patients with a Long Period of Chronic Spinal Cord Injury Present Significant Differences in Circulating CD4+ Foxp3+ CD25+/CD25low Regulatory T Cells Populations

**Figure 1:**
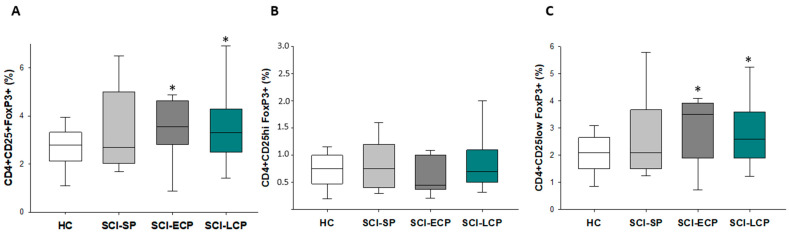
Characterization and percentage of circulating Treg cells according to the presence of specific markers: (**A**) CD4+ CD25+ Foxp3, (**B**) CD4+ CD25hi Foxp3 and (**C**) CD4+ CD25low Foxp3+ Tregs. Healthy controls (HCs); chronic SCI patients with a short period of evolution (1–5 years (SCI-SP)); chronic SCI patients in the early chronic phase (5–15 years (SCI-ECP)); and chronic SCI patients in the late chronic phase (>15 years-(SCI-LCP)). *p* < 0.05 (*).

As a final objective, we aimed to study and characterize circulating Treg cells in patients with chronic SCI using specific markers (CD4, CD25, and Foxp3). In this sense, we observed that there was a significant increase in the amount of circulating CD4+ CD25+ Foxp3+ in SCI-LCP compared to HC (SCI-LCP = 3.8 [2.5–4.3]; HC = 2.7 [2.2–3.3], * *p* = 0.049), and SCI-ECP (= 3.5 [3.2–4.6], * *p* = 0.046). When we considered the level of expression of CD25 (CD25 high -hi- or CD25low), we observed a similar increase in CD4+ CD25low Foxp3+ for SCI-LCP (SCI-LCP = 3 [1.9–3.6]; HC = 2.1 [1.5–2.6], * *p* = 0.049), and SCI-ECP (SCI-ECP = 2.9 [2.2–3.9], * *p* = 0.043), but no variations were observed for CD4+ CD25low Foxp3+ (Figure 1A–C).

### 3.3. Patients with a Long Duration of Chronic Spinal Cord Injury Present a Significant Increase in Circulating CD4+ CD25+ Foxp3+ Regulatory T Cell Populations Negative for CD45RA and CCR7 Expression

**Figure 2:**
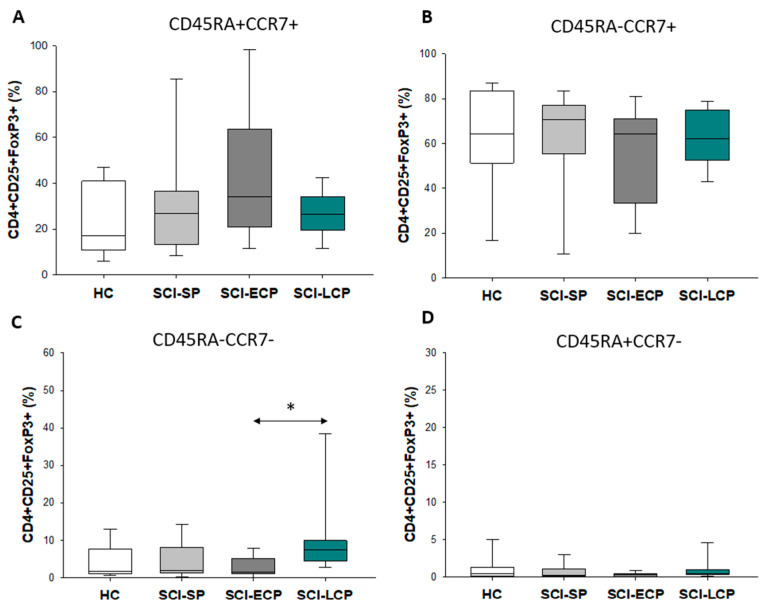
CD45RA and CCR7 expression in CD4+ CD25+ Foxp3+ Tregs. 4 subpopulations are recognized: (**A**) CD45RA+ CCR7+; (**B**) CD45RA− CCR7+; (**C**) CD45RA− CCR7−; (**D**) CD45RA+ CCR7−. Healthy controls (HCs); chronic SCI patients with a short period of evolution (1–5 years (SCI-SP)); chronic SCI patients in the early chronic phase (5–15 years (SCI-ECP)); and chronic SCI patients in the late chronic phase (>15 years-(SCI-LCP)). *p* < 0.05 (*).

On the other hand, we analyzed the expression of the markers CD45RA and CCR7 in the three Treg populations. For CD4+ CD25+ Foxp3+, we only observed a significant increase in CD45RA− CCR7− in SCI-LCP when compared to SCI-ECP (SCI-LCP = 10.1 [4.5–9.9]; SCI-ECP = 3.9 [1.3–6.8]; * *p* = 0.02, Figure 2A–D). 

**Figure 3:**
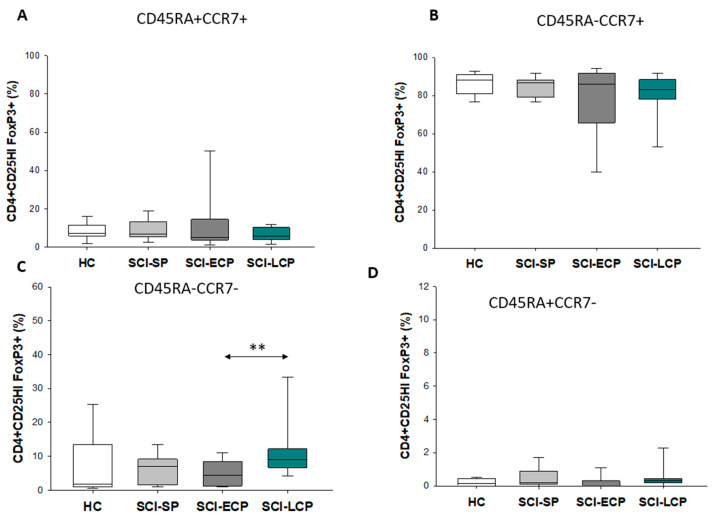
CD45RA and CCR7 expression in CD4+ CD25hi Foxp3+. 4 subpopulations are recognized: (**A**) CD45RA+ CCR7+; (**B**) CD45RA− CCR7+; (**C**) CD45RA− CCR7−; (**D**) CD45RA+ CCR7−. Healthy controls (HCs); chronic SCI patients with a short period of evolution (1–5 years (SCI-SP)); chronic SCI patients in the early chronic phase (5–15 years (SCI-ECP)); and chronic SCI patients in the late chronic phase (>15 years-(SCI-LCP)). *p* < 0.01 (**).

**Figure 4:**
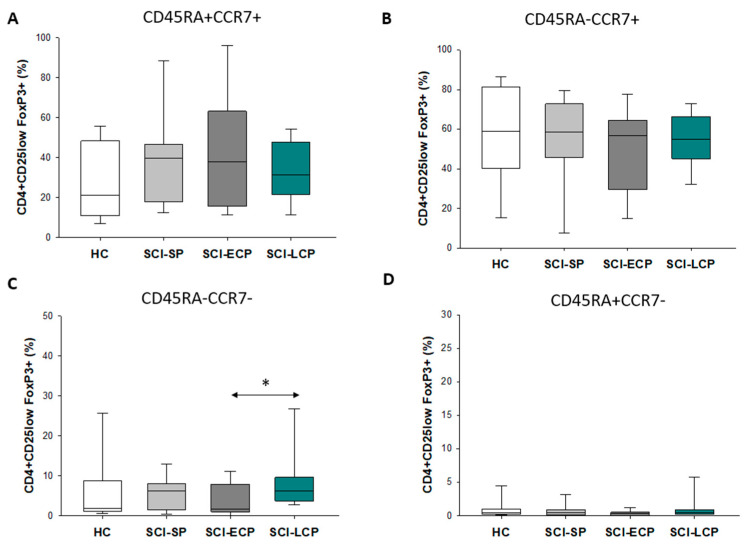
CD45RA and CCR7 expression in CD4+CD25low Foxp3+ Tregs. 4 subpopulations are recognized: (**A**) CD45RA+ CCR7+; (**B**) CD45RA− CCR7+; (**C**) CD45RA− CCR7−; (**D**) CD45RA+ CCR7−. Healthy controls (HC); chronic SCI patients with a short period of evolution (1–5 years (SCI-SP)); chronic SCI patients in the early chronic phase (5–15 years (SCI-ECP)) and chronic SCI patients in the late chronic phase (> 15 years (SCI-LCP)). *p* < 0.05 (*).

The same results were obtained for both CD4+ CD25hi Foxp3+ (SCI-LCP = 13.5 [6.7–12.1]; SCI-ECP = 4.7 [1.4–7.5], ** *p* = 0.007, Figure 3A–D) and CD4+ CD25low Foxp3 (SCI-LCP = 9.8 [3.8–9.1]; SCI-ECP = 4.2 [1.2–7.8], * *p* = 0.045, Figure 4A–D).

### 3.4. Patients with a Long Period of Chronic Spinal Cord Injury Present Statistically Significant Differences in Circulating CD4+ CD25+ Foxp3+ Regulatory T Cells Populations Positive for CCR5 Expression

**Figure 5:**
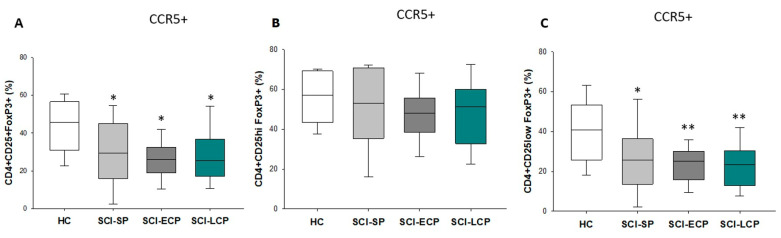
CCR5 expression in (**A**) CD4+ CD25+ Foxp3, (**B**) CD4+ CD25hi Foxp3 and (**C**)CD4+ CD25low Foxp3+ Tregs. Healthy controls (HC); chronic SCI patients with a short period of evolution (1–5 years (SCI-SP)); chronic SCI patients in the early chronic phase (5–15 years (SCI-ECP)) and chronic SCI patients in the late chronic phase (>15 years (SCI-LCP)). *p* < 0.05 (*); *p* < 0.01 (**).

However, when we considered the expression of CCR5, we observed that there was a significant decrease with respect to HC in the expression of this marker in patients in the SCI-LCP (SCI-LCP = 29.4 [17–36.8]: HC = 43.7 [31.1–55.6], ** *p* = 0.006), SCI-ECP (SCI-ECP = 25.5 [20–30.8], * *p* = 0.04), and SCI-SP (SCI-SP = 30 [19.2–42.9], * *p* = 0.037) groups. These results were the same for CD4+ CD25low Foxp3+ in the case of SCI-LCP (SCI-LCP = 24 [12.8–30.3]: HC = 40.7 [26.6–51.6], ** *p* = 0.002), SCI-ECP (SCI-ECP = 23.4 [16.4–29.2], ** *p* = 0.01), and SCI-SP (SCI-SP = 26.4 [16–34.8], * *p* = 0.039), but not for CD4+ CD25hi Foxp3+ (Figure 5A–C). 

## 4. Discussion 

After the initial trauma, a time-dependent multiphasic response of cellular inflammation occurs in the injured spinal cord, with a maximum peak of T cell infiltration at day 9 post-injury [27]. In chronic stages, T cell recruitment and inflammatory reactions in the spinal cord persist, promoting both protective and pathogenic mechanisms. Tregs are a subgroup of CD4 T cells involved in the suppression of inflammatory responses [36]. After SCI, Tregs seem to promote neuroprotective effects and remyelination, although their levels may be determined by temporal and spatial factors, as a balance between Tregs and effector T cells is critical for maximizing spinal cord repair [28,37]. In more detail, a reduction in Tregs in the early stages might favor neurological recovery and repair, whereas decreased levels in the subacute or chronic stages may negatively interfere with tissue remodeling [38]. Monahan et al. [31] observed that patients with chronic SCI exhibited elevated frequencies of CD4+ Tregs, particularly in the CD25+ CD127loCCR4+, CD25+ CD127loHLA-DR+, and CD25+ CD127loCCR4+ HLA-DR+ populations, and hypothesized that these changes might be relevant in explaining the infection susceptibility among this population as well as other manifestations of immunosuppression and immune system dysfunction. Similarly, we demonstrated that patients with chronic SCI and a long period of evolution (SCI-ECP and SCI-LCP) display a differential proportion of circulating CD4+ CD25+/low Foxp3+ Tregs than healthy controls. However, this pattern changed regarding the expression of CCR7 and CD45RA, as we observed that the SCI-LCP patients exhibited a noteworthy increase in CD4+ CD25+/hi/low Foxp3+ CCR7− CD45RA− Tregs when compared to SCI-ECP patients. Finally, we also determined that SCI-LCP, SCI-ECP, and SCI-SP patients displayed significant decreases in CD4+ CD25+/low Foxp3+ CCR5+ Tregs in comparison to the healthy controls. Hence, considering our results, it is feasible to suggest a potential role of these changes in the evolution and progression of chronic SCIs. 

Firstly, we were able to characterize an increased percentage of CD4+ CD25+ Foxp3 regulatory T cells in patients with SCI-ECP and SCI-LCP in comparison with their healthy controls. This relationship was equally observed for CD4+ CD25low Foxp3, but not for CD4+ CD25hi Foxp3, demonstrating the influence of this marker on the samples which were studied. Foxp3+ Tregs are recruited by monocyte-derived macrophages to the injured spinal cord parenchyma at the subacute/chronic phase, fulfilling a critical role in tissue repair [28]. CD25 expression is thought to be one of the ways by which Treg suppresses the proliferation of effector T cells, acting as a sink for IL-2 [39]. An increase in Tregs, characterized by lower expression of CD25, may thus be an indicator of a reduced capacity to modulate effector T cells in patients with SCI-ECP and SCI-LCP, which may have detrimental consequences for these subjects. On the other hand, previous works have reported a significant expansion of CCR4+, HLA-DR+, and CCR4+ HLA-DR+ Tregs in patients with chronic SCI [31]. Despite the fact that the authors did not classify these patients according to time, the average time since initial injury was designated as between 14.3 and 19.7 years, hence supporting our results that patients with SCI-ECP and SCI-LCP display significant alterations in the immune Treg compartment. The exact roles of Tregs in SCI are complex, and the functional implications of Treg expansion are not fully understood. Moreover, it is still unknown whether changes in Tregs cause immune dysfunction or whether they expand in response to altered immune environments [7].

To deepen our knowledge of the characterization and phenotypes of the altered Tregs, we aimed to study specific markers of these cells, selecting CD45RA, CCR7, and CCR5. Interestingly, we observed an expansion of CD4+ CD25+ Foxp3 CD45RA− and CCR7− regulatory T cells (called induced Treg cells) only in the SCI-LCP group, independently from low or high expression of CD25. This might be an indicator that the expression of both markers is transiently lost in Tregs with the progression of SCI. CD45RA− Tregs appear to present a reduced immunosuppressive capacity compared to those with CD45RA+ [23], and the absence of CD45RA is associated with the loss of CCR7 [40]. In turn, the absence of CCR7 expression is also related to the reduced function of CD4+ CD25+ Tregs [24]. Intriguingly, previous works have noted that CCR7 expression suppresses the immunodeficiency phenotype by activating the chemokine signaling in T follicular helper cells after SCI [41]. Thus, it is likely that the expansion of induced Tregs in the SCI-LCP group may be related to a loss of functionality, contributing to the immune dysfunction observed in these patients. On the other hand, we observed that there was a significant decrease in the percentage of CD4+ CD25+/low Foxp3+ CCR5+ in patients with SCI-SP, SCI-ECP, and SCI-LCP in comparison to the healthy subjects. Prior works have claimed that CD4+ CD25+ CCR5+ Tregs promote the further recruitment and immunosuppressive actions of Tregs [25]. Moreover, the blockade of CCR5 seems to promote enhanced polarization to anti-inflammatory (type 2) macrophages after SCI, improving locomotor recovery in mice [42]. The decrease in Tregs expressing CCR5 in a time-dependent manner may be consistent with the previous results, which support that despite the increased proportion of CD4+ CD25+/low Foxp3+ Tregs in patients with SCI-ECP and SCI-LCP, these cells can be functionally affected, with potential implications for their ability to modulate other immune populations. 

Our study has some important limitations that should be further addressed in future studies. For example, we did not consider the different manifestations of immune dysfunction in the studied patients, and we were not able to include different individual variables or the presence of certain comorbidities to cluster among SCI patients. In our sample, approximately 70% were male, and a similar percentage exhibited SCIs above T6. Thus, our results might not be suitable for extrapolation to the totality of the SCI population. Further complementary techniques should also be considered for future studies. Conversely, our study also presents some important strengths, such as the relatively high sample size in comparison to similar studies; thus, our results seem to be robust and consistent within SCI patients. Furthermore, a broader characterization of T cells and Treg phenotypes will be of great aid in gaining further insights into the immune dysfunction observed in the SCI population.

## 5. Conclusions

**Figure 6:**
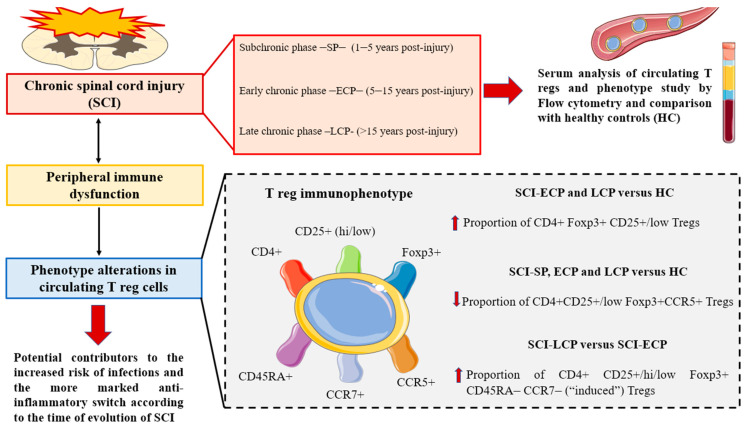
A summary of the main results compiled in the present work. Herein, the main phenotypic changes observed in patients with chronic SCI are summarized. Overall, there is a need for a deeper characterization of the immune dysfunction occurring in these patients, especially considering clinical correlates and variables.

In the present work, we demonstrate that patients with SCI have significant changes in circulating Tregs depending on the time since the injury. Specifically, patients in the SCI-ECP (5 to 15 years post-injury) and SCI-LCP (>15 years post-injury) groups seemed to present an increased proportion of CD4+ CD25+/low Foxp3+ Tregs in comparison to healthy subjects. In addition, a decreased number of these cells expressing CCR5 was observed in the SCI-LCP, SCI-ECP, and SCI-SP subjects (<5 years). Concomitantly, an increased amount of CD4+ CD25+/hi/low Foxp3, with negative expression of CD45RA and CCR7, was observed in SCI-LCP patients when compared to those in the SCI-ECP group. Collectively, as shown in Figure 6, the altered Treg cell populations observed in patients with chronic SCI may aid in explaining the immune dysfunction reported in this group of subjects and how the time since initial injury may drive this dysregulation. 

## Data Availability

The data used to support the findings of the present study are available from the corresponding author upon request.
